# Towards an Integrated Care Organisation from a CEO Perspective

**DOI:** 10.5334/ijic.5559

**Published:** 2020-08-17

**Authors:** Gerardo Amunarriz, Henar Alcalde-Heras

**Affiliations:** 1 Matia Institute, ES; 2 Deusto Business School – University of Deusto, ES

**Keywords:** organisational integration, leadership competences, integrated care, person-centred care, management, culture change

## Abstract

Many experimental projects towards Person-Centred Care (PCC) are successful in the early stages, but founder when the attempt is made to scale them up to encompass the whole organisation. This case study therefore focusses on one manager’s attempts to extend the successes of a preliminary project ‘Etxean Ondo’ that aimed to provide adequate support for the elderly living at home or in nursing homes, as well as for their families and care professionals. Through in-depth interviews with stakeholders, this qualitative study, based on Grounded Theory, sets out to analyse which behaviours, attitudes and values on the part of management appeared to favour full-integration of PCC in this wider context. Analysis of the data gathered allowed the researcher to generate an experimental case model which suggests how the extrinsic, intrinsic and transcendent motivation of stakeholders can be aligned with the goals of upper management to promote full-integration of PCC in such a way as to generate trust, increase participant engagement and create a win-win situation for all. Whilst this is clearly an experimental project, it is hoped that the model provided may prove helpful to other researchers and managers interested in pioneering this type of comprehensive organisational strategic change towards integration.

## Introduction

Research suggests that successful integration of PCC depends essentially on the routines and attitudes of frontline staff, especially in their relationships and interdependent teamwork [1,2,3,4]. This is consistent with the positive results of Etxean Ondo, an experimental project [15] that aimed to provide adequate support for the elderly living at home or in nursing homes, as well as for their families and care professionals. This support and care were based on a coherent set of methods designed to improve communication and collaboration within and between the healthcare system, long term care-systems and the community.

Hence, any attempt to progress towards generalization and consolidation in line with PCC [6,7] requires not only clinical and professional integration but also organisational and systemic integration [8]. According to Leutz [1] “full integration” implies building a new relational framework [2] based on the concepts of interdependence and decentralized teamwork [9,10]. These statements are consistent with relevant models such as DMIC, RAINBOW, PRISMA, KAISER, PACE, CCM, or MBQA, which recognise the centrality of a bottom-up logic, micromanagement flexibility, and close case management [11].

Successful integration of PCC also depends on informal aspects [8,12] that are directly influenced by the recognition of the subjectivity and diversity of persons, as well as intangible factors like knowledge and personal motivation [13]. These experiences generate learning [14,15,16,17] and changes in personal beliefs and values, leading to changes in people’s criteria for decision-making [18] which can foster or impede the process of change [15].

When managers try to integrate these “informal” and relationship-oriented realities in practice [2] using the revised models [19,20], they frequently encounter difficulties which arise from contemporary organisational culture. The current organisational paradigm bases its quality, wellbeing, and security exclusively on the stability and static control of formal structures and protocols that use top-down approaches and avoid all these intangible realities that are tough to manage and measure [12,21]. However, managers ignore the dynamic nature of the process of change at their peril, as any management response which focuses purely on static protocols will be unable to respond to the changing needs of stakeholders [22].

Research consistently highlights management as either an enabler or barrier to successful integration of these “soft” and spontaneous realities and the generation of integrated organisations [23,24,25,26,27,28,29,30]. According to Miller and Stein [12], management for integrated care remains an underdeveloped concept and practice. Specifically, the lack of conceptual clarity among managers regarding how to scale, transfer and sustain this process [12,31] effectively and confidently [21] towards the organisational integration of PCC [8,11,23,32,33] remains a significant barrier [12,27,28,29,30,34,35,36]. More research is urgently required to understand how to support managers so that they can achieve changes in practice and culture.

The objective of this article is therefore to identify, from the perspective of the CEO, the organisational drivers and management skills that steered the positive strategic change process of a pioneering integrated care organisation in the Spanish Basque country towards its full integration [5,37]. This study is clearly of an exploratory nature and still in progress, but it is hoped that the findings will provide food for thought for other managers concerned with achieving full-integration of PCC across the larger organisation.

## Case study background

### Matia Foundation

Matia Foundation is a non-profit organisation founded in 1889 and located in the Basque Country (Spain). Table 1 shows key figures for the Foundation. The main researcher and author of this paper is the CEO of Matia foundation who has been involved in the design, planning and implementation of the Etxean Ondo project from its inception until now.

**Table 1:** Matia Foundation main figures.


Turnover	52 M€ —> 85% comes from different public administration, bundled, act and cash payments
Patients/users	+30,000/year
Direct Employees	1,428
Indirect Employees	2012 (14%) –> 2016 (9%) —> 2019 (8%)
Volunteers	3,418/168 organizations
Health Services	Geriatric Hospital (103 beds), average stay: 24 days
	Geriatric outpatient consultations
	7 Rehabilitation centers
Long Term Care	8 Nursing homes (1,072 beds)
	7 Day centers
	Two apartment blocks
	Home Care in the province
Community	Age friendly activities in 70 towns/locations in the Spanish Basque Country


### The catalyst for change and the ‘Etxean Ondo’ PCC project

In 2010, the social policy department of the Basque government of Spain published a study about the living conditions of elderly people in the region [38]. One of the main conclusions of the study was that older people wanted to live in their own homes, and where this was impossible to live in conditions as home-like as possible. The study also showed that the care system was highly fragmented and was prioritising the needs of service-providers over those of service-users. Above all, the focus was on meeting the practical, physical requirements of service-users while ignoring their emotional, psychological and spiritual needs, providing a ‘one-size fits all’ care package which failed to take into account the diversity of individual needs.

In response to these findings, the Department of Employment and Social Policy of the Basque Government and Matia Foundation together launched a EUR 7 million pilot project called ‘Etxean Ondo’ [5] (from the Basque for ‘at home, and well’), in coordination with the Basque Health System. These two institutions worked in close collaboration with the municipalities and the district’s public health care services at a policy level, as well as with other health and social agents. The aim of the project was to provide adequate support [39], in a more sustainable and cost-effective way [2], for the elderly living at home or in nursing homes, as well as for their families and care professionals [23], by changing to a model of Person Centred Care [2].

Etxean Ondo proposes a single entry point; a case management methodology and an individualised service plan, all of which respect the personal dignity, rights, interests, and preferences of the service user. The main organisational mechanisms used were the creation of self-management and interdependent teams which provided continuous care, as well as the involvement of all community stakeholders, including informal carers and volunteers. Social workers were assigned the role of case managers who interacted with primary care nurses, who in turn interacted with GPs. These professionals worked together to meet both social and health care needs.

In addition, a new role was created for the coordination of care teams. During the experimental phase these coordinators played an essential role in ensuring the flow of information and de-centralisation of decision-making. These unit coordinators had a dual role: each coordinator was responsible for his/her own team or unit, and at the same time was a member of the next level of team management. This allowed the bottom-up transmission of needs and top-down flow of resources, decision-making delegation and support. It has been observed that team stability and small size [8,9,10,11,33,40] are essential for learning and generating trust, progressively generating a virtuous spiral leading to greater empowerment and improved dialogue. Each team therefore had structured teamwork spaces in order to encourage collective processes of action and reflection for learning and sense-making [31,41].

The results of this experimental phase of Etxean Ondo were recognized by the Strategic Intelligence Monitor on Personal Health Systems Phase 3 which selected the project as a case of good practice from the 24 finalists in the final round [5]. Measurable results of the experimental project included a reduction in hospitalisations in terms of the average length of stay and emergency visits. Furthermore, the functional status and health outcomes of the elderly improved, together with the quality of life and satisfaction of patients and carers alike.

In terms of the financial outcome of the experimental phase, the reduction in indirect labour costs since the beginning of the project has been significant. In 2012, indirect personnel accounted for 14% of staff at the Foundation, while at the end of 2019 only 8% were indirect personnel. This reduction has allowed these resources to be redirected towards more direct care-givers (+4%), technological investment, and R&D projects.

All of these promising results of the Etxean Ondo project enabled us to verify the effectiveness of the proposed paradigm, supporting the expansion and consolidation stages [6] between 2016 and 2021. The findings also support the development of an exploratory research study focused on understanding the transformation process of the organisation from a managerial perspective [12,27]. The rest of this paper reports on the findings of this process of expansion and consolidation which began in 2016.

### Methods

The constructivist approach based on Grounded Theory has been accepted as an appropriate methodology for research into complex social realities [12,27,32,42,43], combining systematic inductive, deductive, and verification approaches to construct mid-range theoretical frameworks [43,44,45].

The data presented in this article was extracted from two different sources. The first source consists of two studies carried out in 2016 and 2017 in four Matia Foundation centres. These studies analysed the changes in clinical and professional integration from the perspective of teams of direct care professionals via discussion groups led by an external researcher [44] with 32 people in total (Table 2).

**Table 2:** Focus group data collection details.

Date	Setting	Number of Beds	Number of years this setting has been part of Matia	Participation in Etxean Ondo project	Focus group participants	Focus group size	Level of integration

**1st October 2016**	**Nursing Home F**	**88**	20 years	yes	Director + multidisciplinary team + caregivers	9	Full
	**Nursing Home L**	**143**	12 years	yes	Multidisciplinary team + caregivers	7	Coordination
**2nd June 2017**	**Nursing Home R**	**120**	120 years	yes	Caregivers	9	Coordination
	**Nursing Home P**	**103**	2 years	no	Caregivers	7	Linkage

The results of these discussion groups helped identify the main changes experienced in the care routine of the different centres and services under study. This allowed us to plan a second research phase in order to gain a deeper insight into the main concerns identified in phase one. Using in-depth interviews [12], the CEO and main researcher interviewed operational and strategic managers in key positions within the organisation. This phase aimed to identify key organisational drivers for PCC implementation [42,46]. Data collection took place from June 2017 to September 2018.

The main researcher played a dual role, since he is the CEO of the organisation under analysis [43], and therefore, has been directly and personally involved in the integration process which began in 2011. Of course, there are risks associated with this dual role. The CEO is likely to encounter role conflict and get caught between loyalty tugs and identification dilemmas [47]. Similarly, as posited by Coghlan [43], undertaking a research project in one’s own organisation is political and might even be considered subversive. Moreover, there is a risk that insider researchers might suffer from “lock-in” because they accept certain situations and do not consider alternative frameworks [47]. Finally, the target group may be suspicious of an insider researcher who is also the manager of the organisation, because of his or her hierarchical position, and organisational resistance could potentially emerge due to conflicts of interest.

Despite these obvious risks, the unique position of the CEO/researcher allows him to develop insights into the process from a management perspective, and his involvement in in-depth research and analysis can provide a level of reflection rarely experienced by busy executives. Such an approach is compatible with the principles of grounded theory, providing an experiential understanding of the issues at stake, as well as organisational idiosyncrasies and milestones achieved.

The data analysed in the next section was obtained through 11 in-depth interviews led by the CEO. The interviewees were selected to represent diversity in terms of the following criteria (Table 3) [1,42,43,48]:

1. Covering different positions, from care-givers to Foundation board members
2. Length of service
3. Educational background (health, social, and management)
4. Experience in PCC integration
5. Oral communication skills
6. Experience in R&D projects

**Table 3:** Details of the in-depth interview data collection.

Data Source	Position	Length of service	Setting	Educational background	Experience in PCC integration	Oral communication skills	Previous experience in collaborating in R&D

**In-depth interviews from June 17 to September 18**	**Social Worker**	37 year	Hospital	Social	10 year	High	Often
	**Caregiver**	38 years	Nursing Home B	Social	7 years	Low	Rarely
	**Psychologist**	20 years	Nursing Home L	Social	8 years	High	Often
	**Nurse**	30 years	Hospital	Health	10 years	Medium	Rarely
	**Center Manager**	19 years	Nursing Home F	Social	8 years	High	High
	**Procurement officer**	35 years	Support	Economics	8 years	Medium	Never
	**Controller**	1 year	Management	Economics	0 years	Medium	Never
	**Accountant Officer**	40 years	Support	Economics	10 years	Medium	Never
	**Chair Woman**	16 years	Board of trustees	Humanities	4 years	High	Often
	**Vicepresident**	20 years	Board of trustees	Economics	6 years	High	Rarely
	**Secretary**	6 years	Board of trustees	Administration	2 years	High	Rarely

The interviews were conducted in the following stages:

- “Ice-breaker”: an initial step explaining the purpose of the research and dealing with the first work experiences of the interviewees within the organisation. (10 to 15 minutes).
- “Data mining”: focused on analysing the situation at that moment and the changes observed in the PCC integration process from the participant’s individual perspective (30–45 minutes).
- “Positive closure”: evaluating what did or did not work or what would have been desirable in the PCC integration process (10 to 15 minutes).

All the interviews were recorded, transcribed and analysed, both manually and using Nvivo 12 software. The process took place sequentially such that the analysis and coding of each interview was carried out before the following interview. In this way, the findings arising from the first interviews were compared with the following ones following the methodology of Grounded Theory [44,45]. This process made it possible to identify new questions and follow up on them in successive interviews. The analysis and observation process lasted 15 months and was completed in September 2018, when the process of research and analysis was no longer yielding fresh insights.

### Emerging categories

Through an inductive process of categorisation based on the transcripts and notes taken during the interviews, 167 codes were generated. These codes were confirmed through constant comparison using NVivo 12 software to add validity to the findings. The final outcome of the inductive process generated 9 case categories (Table 4). A further series of ‘support’ categories were identified (Table 5) based on intrinsic and extrinsic motivators, and fear of change.

**Table 4:** Description of the organisational categories emerging from data analysis.

Category	Description	Citation	Subcategories

**Mission of service**	This category represents the organisational objective, i.e. exploring what people need as well as the level of satisfaction of the participants, both care recipients and care providers, in order to achieve their well-being. Autonomy is imbricated in the individual’s recognition of a collective purpose of service and in his/her identification with it, which gives the participants in this service a sense of purpose.	*“The fundamental orientation of our service… is towards the person.” “Because we come to work to take care of their needs, we also have to take care of our own, that is, physically, mentally and emotionally”*.	Well-being, certainty, security, autonomy.
**Experiential learning**	PCC incorporates new ways of working which entail new technical, interpersonal and intrapersonal skills to manage situations of great emotional burden and stress. The key lies in facilitating experimentation by encouraging collective reflection and creativity for solving the problems in the work itself.	*“… what do we need then? Skills”. “The concepts are there, but what really happens is that you have to develop them. And it is practice itself… There has been blood, toil, tears and sweat! And much effort and sacrifice…”*	Core competences (technical, interpersonal and intrapersonal), collective reflection, personal resignation and effort.
**Participatory Person Centred planning**	Acts as a lever that turns the mission of service into action. Focus on the exploration of needs, consensus in prioritising goals and the design of action. As all these take place at the same time, this is a multilevel structure which connects people with the organisation and the environment.	*“because we have been given the autonomy and freedom to be able to develop our management plan.”*	Needs analysis, consensus, prioritisation towards mission.
**Policies and information systems**	This category encompasses a whole structure of policies, information systems, standards, procedures and protocols to promote communication and participation, avoiding individualism. PCC requires flexibility and adaptation, and protocols and systems become “containers” of collective learning. Democratic IT systems are mandatory	*“We are working with people, and if you want the model to work, everything needs to be integrated, and no aspect can be shut off from the others” and therefore “we need to have a complete view of the organization, a balance among all our needs: schedules, organisation, personal functioning, delegating tasks, and autonomy…”*	Share-decisions to avoid opportunism, removing protocols, open information systems.
**Formal organisational structure**	Represents the functions and roles assigned to people to facilitate the delegation and information flow of formal power in decisionmaking according to required level of participatory planning. Critical aspects: the stabilisation of the teams, grouping people into small groups of up to 16 members and “home environnent”.	*“Before 1 didn’t mind being with XXX, or with YYY… because we were 30 or 40 colleagues and we would be always changing. Now there are just are seven team members who meet and decide”*	New professional roles, structured teamwork spaces for reflection, no rotation (team stability).
**Informal organisational structure: “Diversity”**	Recognition of the uniqueness of each individual. The deepening of relationships has allowed people to become visible as individuals and not only through the role they play. This fosters generative dialogues that boost creativity. The recruitment of new team members is a critical process.	*“Well, we already saw that so much protocol and corporate rigidity and that way of working were not appropriate for taking care of people or their feelings”*.	Equity, recognition of personal diversity, source of knowledge.
**The relational style: problemsolving approach**	Dialogue and fluency of information among the participants. Accompanying participants in situations of great dependence and fragility is a complex duty that requires continuous coordination of the care services and actions of the different participants, within each team, across work shifts, and across the whole organisation. This calls for the integration of the different capacities through greater communication, which is given different meanings:	*“We talk a lot, share a lot, listen a lot” “… we are like facilitators of people’s lives.” “having no secrets”, “dealing with slacking”, “being informed of everything that happens”, “being coordinated and having the same objective” or “coordinating the care provided between shifts with fluent communication and informing about incidents each day”*.	Active listening, problem solving approach, accompanying
**Cooperative culture: “based on mutual trust”**	Dialogue and fluency of information among all the participants in order to solve a complex duty that requires continuous coordination of the care services.	“Until we learned to respect and became aware that, actually, if we rejected something, it was because we didn’t understand it…” “Well, actually they don’t work against me, in fact, we work together well!”	Inclusion, diversity as a value of the person, intrinsic dignity, identification, being part of something bigger, affective cohesion.
**Personal leadership**	Personal leadership is empowerment, discipline and self-control, regardless of whether there is formal power. Leadership is the result of a process of renunciation and learning, where people and teams are the priority.	“Leadership has to do with self-understanding and valuing what you have, and the feeling of connecting with something bigger, with a greater being than yourself.”	Motivational quality (ability to prioritise common good), empowerment, self-knowledge.
		“Leadership skills have to do with autonomy, don’t they? We have the power now, so let’s see how we use it.”	
		“You have to empower the people, which means that they are going to decide, my responsibility towards people is more informal than formal. That’s what matters to me!”	

**Table 5:** Description of emerging case support categories.

Category	Description	Citation	Subcategories

**The external environment**	The category ‘external environment’ includes all those actors and aspects that are beyond the control or management capacity of the organisation. This category can have both a positive and negative effect on the functioning of the organisation given that it operates in the economic, social and political dimensions in which it is immersed.	*“Our dependency; we depended a lot on financing and economic-political agreements. (…) At the family level we have a very protectionist culture, and that leads to the need for control.“*	Families, society, public administration, health system, long term care sector, community, ecosystem agents.
**The internal environment: “personal scope”**	This category includes all those aspects that are inside the participants themselves and within their personal environment. Especially relevant are their personal beliefs and experiences which have a direct effect on the activity of the organisation but which cannot be handled by the organisation itself. These aspects are also influenced by the external environment.	*“Well, I was very young, and I was very enterprising. I was deeply committed, from an ideological point of view…” “If I have problems at home, I get emotionally affected, and bring this state of mind to work”*.	Personal beliefs, values, background and experiences.
**Personal motivation**	This category represents the goals of human action; what the participants want to achieve through their action to solve their dissatisfactions. This is considered a support category because it is linked to people and not directly to the organisation. Nevertheless, it is the category through which we can connect people with the organisation through the decision-making process, given that personal motives are present both in the design of the formal organisation and in the informal relationships which arise in the organisation. These personal motives directly affect group performance and effectiveness.		
**Extrinsic motivation**	These aspects are external and take the form of material compensation or intangible rewards such as prestige, recognition or praise. They respond to needs for security, uniqueness (recognition), belonging (v. isolation) and fun.	*“I worked for the money, of course. I wanted my wages and that was all”*.	Bio-material-physical needs, perceptions, security, uniqueness, belonging, fun.
**Intrinsic motivation**	These motives are internal goals that each person hopes to achieve as a result of an action. It is therefore a question of knowing one’s own personal needs regarding goals and personal growth. They respond to the need for personal improvement.	*“Right now I’m very motivated, because I still have everything to learn and that way I will be able to do new things, and this makes me happy”*.	Psycho-cognitive needs, personal challenge.
**Transcendent motivation**	These motives are linked to the personal satisfaction that comes from caring for other people or supporting the team (care recipients, participants, family etc.), in other words, the personal satisfaction of contributing to the collective self and feeling part of something. They respond to the need to contribute to the common good.	*“I’m motivated by people, yes, people, I like dealing with people… learning about their life experiences… I’m not looking for more pay, I don’t want to be the boss, nor have a higher status and so on, just feel fine supporting.”*	Socio-affective needs, sense of contribution, sense-making.
**Fear of change**	This category emerges as a living code in the narratives collected. Changes that are not in accordance with current skills, lack of information or expectations of participants can cause fear, resistance, anger, flight response, and demotivation, in short, ineffectiveness and barriers.	*“There was a vision, a style of management… And we had to stick to the protocol. We took a macro approach towards everything, which I think was not very realistic, because we didn’t have the appropriate skills. And we were very scared…” “Something that is never talked about is that it is normal to be afraid, to be afraid of change and of the unknown. Because… if anyone gets angry, cries, or just ignores everything… there is a reason for this and the reason is fear” “I find this resistance to change very frustrating. It takes a lot of determination to overcome it. Change is difficult”*.	Fear, resistance, anger, flight response, demotivation, sadness, fear of failure, flight.

All of these categories are described in the respective tables and include typical supporting statements collected from the discussion groups and in-depth interviews.

The uniqueness and diversity of individuals render approaches based only on standardised protocols ineffective. Hence, the reasons why people make decisions and their drive towards personal needs satisfaction have come to constitute the dominant category in the experimental case model (Table 4). In other words, the model is person-centred, deriving from individual needs and purposes.

This process of analysis has therefore identified three types of motivation: extrinsic, intrinsic and transcendent (Table 5). These different reasons explain why people seek to satisfy some needs and not others, and therefore prioritize some behaviours and not others. These categories serve as the guiding principle in the constructivist process, connecting participants, their decision-making and behaviours with organisational reality. In other words, they allow us to connect the participants’ subjective needs and behaviours with the aims of the organisation.

### Exploratory case model

Having defined the twelve case categories, this section focuses on identifying the relationships among them to propose an experimental model which could help managers identify the key organisational levers that may be used to promote a fully integrated PCC paradigm.

The relationships among the case categories were obtained with NVivo 12 software, using built-in algorithms which compare frequencies and meanings. Table 6 shows the relationship between the nine organisational categories, including a measurement of the relative relationship intensity between them.

**Table 6:** Emerging relationships.

	A: Formal structure	B: Participatory planning	C: Policies & systems	D: Experiental learning	E: Informal structure	F: Relationship style	G: Personal leadership	H: Cooperative culture	I: Mission of service

1: Formai structure				10		8		9	
2: Participatory planning									8
3: Policies and systems							8		
4: Experiental learning	10					8		8	
5: Informal						8	10		
6: Relationship style	8			8	8				12
7: Personal leadership			8		10				10
8: Cooperative culture	9			8					10
9: Mission of service		8				12	10	10	
Sum	27	8	8	26	18	36	28	27	40

“Mission of service” is the category which relates most strongly with other categories, with four direct relationships to: “participatory planning”, “personal leadership”, “cooperative culture”, and “relationship style”. The dominance of this factor confirms the impressions garnered through the collected narratives. “Relationship style” also stands out as highly important.

“Personal leadership”, “cooperative culture”, “experiential learning” and “formal structure each relate to three categories. It is interesting to note that, of these six categories with high or medium intensity inter-relationships, only “formal structure” is directly related to management. The rest belong to the informal context and depend on the free-will of participants.

The category “informal structure” shows medium-low relationship intensity, relating to only two other factors: leadership and learning. This makes sense, as these factors are intrinsic to individuals. Finally, “participatory planning” and “policies and systems” only relate to one other factor each. These two categories belong to the formal context. This is a finding which needs to be validated by further studies to confirm that it is due to the greater importance of informal aspects in an integrated organisation.

A second step in the inductive process involved identifying the potential relationships between the nine organisational categories and the external and internal environments. The results show that both “mission of service” and “participatory planning” were oriented towards the external environment, while “informal structure” focused on the internal environment of participants.

Finally, the relationship between the nine organisational categories and personal motivations was analysed to see if any pattern emerged.

The Nivo 12 software reveals (Table 7) an interesting pattern which groups the nine organisational categories according to three different realities.

**Table 7:** Relationships between types of motivation and organisational categories.

Motivation style	A: Formal structure	B: Participatory planning	C: Policies & systems	D: Experiental learning	E: Informal structure	F: Relationship style	G: Personal leadership	H: Cooperative culture	I: Mission of service

Extrinsic	3	6	2	0	1	0	0	0	1
Transcendent	0	0	1	0	0	1	6	6	4
Intrinsic	0	1	0	4	5	4	0	0	0

**Figure 1:**
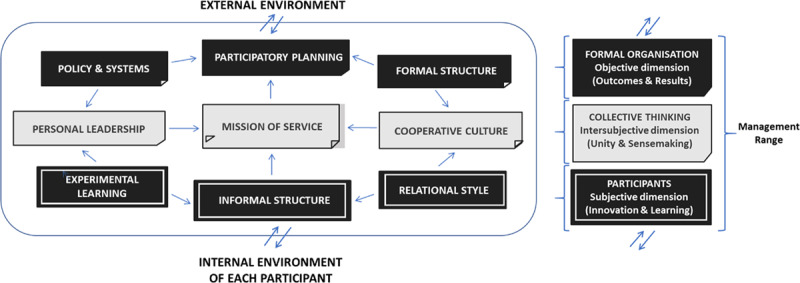
Exploratory case model.

The first is linked to extrinsic drives and responds to the objective aspects of the formal organisation, including “formal structure”, “participatory planning”, and “policies and systems”. The second is linked to intrinsic motivation and refers to the *subjective dimension of participants*: “experiential learning”, “informal structure”, and “relationship style”. Lastly, a third reality emerges linked to transcendent motivators, representing the *intersubjective area* of collective thinking which gives meaning to the “collective self”. This dimension appears to consist of “mission of service”, “personal leadership”, and “cooperative culture” (Figure 1).

Figure 1 offers a model for the integration of all the findings of the inductive process described above. This includes a combination of the results and relationships obtained by Nivo 12 with the manual “records” and thoughts collected during the interviews by the insider-researcher. The experimental case model proposes an integrated organisation structured according to individual motivations (extrinsic, intrinsic and transcendent) that seek to satisfy personal needs (bio-physical, psycho-cognitive and socio-affective). This integrated organisation strives to link two environments: the external environment, which is unique and tangible and affects all levels and participants within the organisation, and the internal environment, which is an internal, intangible environment unique to *each participant*.

The subjective dimension relates to the participant’s unique value as a source of organisational knowledge-generation and innovation based on experiential learning and relationships with other participants (Table 6). This new source of knowledge exponentially increases innovation when it is of a cooperative nature. In addition, these intrinsic motivators (Table 5) make it possible to connect the organisation with the internal environment of each person, especially with those changing values and beliefs that are outside the control of the organisation and yet directly influence the behaviour of the individual.

At the same time, formal organisation seeks the highest cost-effectiveness to achieve the best possible outcomes from the external environment. The participatory planning of users and different care agents provides the collaborative spaces needed for person-centred policies.

The experimental model shows a third, intermediate reality: the collective thinking that makes it possible to integrate the system through the organisational development of mission, cooperative culture and personal leadership (Table 4). This dimension is linked to transcendent motivation (Table 5) and aims to create affective unity through a collective sense-making process in order to promote the wellbeing of all participants. This seems to be the key to generating both a sense of personal safety and organisational flexibility during the integration of PCC.

### Emerging dynamics of the experimental model

The final stage taken to understand the dynamics of strategic change involved comparing the support category “fear of change” (Table 6) with other case categories using Nvivo 12. The results show that only the categories “extrinsic motivation” and “formal structure” are related to this fear.

Both these categories belong to formal organisation. These relationships suggest that, when the process is managed in an extrinsic way (top-down), resistance emerges, following an action-reaction pattern. This resistance generates defensive positions: uncertainty arises, with the suspicion that change may be positive for the organisation but will probably have a negative balance for the individual. These findings are consistent with what was observed in the interviews and discussion group transcriptions showing the correlation between fear and mistrust in management.

Fortunately, the interviews suggested that these fears can be overcome when affective cohesion due to transcendent motivation mobilises the team. When participants perceived the team leader’s predominant motives to be transcendent (concerned above all with furthering the ‘greater good’ rather than focusing on the profit motive, etc.), then cooperation emerged from the feeling of mutual trust and being “part of” something bigger. Thus, this positive pattern for change seems to be grounded in a feeling of contribution and sense-making.

### Emerging implications for CEOs and Change Management

These exploratory results suggest that collective thinking has the potential to be the cornerstone of integration. Such effective collective thinking can be promoted through the consistent management of the nine organizational levers identified above, providing a more flexible but also a stronger basis for innovation than traditional models based solely on the static control of the formal organisation. In other words, collective sense-making towards mission and needs seems to foster intrinsic motivation and generate affective cohesion among participants, leading to creative, collaborative teamwork, learning and innovation.

In fact, the data appear to confirm the findings of Miller et al. [49]: when the prevailing collective thinking of a given setting is directed towards short-term outcomes, the director acts as a manager or transactional leader prioritising the objective dimension. In this case, as observed in centre P, caregivers know where and when they can coordinate with other professionals and services if the circumstances require it, but each one takes care of his or her own formal role and technical competence, prioritising basic physical needs to the exclusion of affective, psychological and other necessities. Under these conditions the level of integration is close to “linkage” as described by Leutz [1].

In contrast, the experience observed in centres L&R of this study appears to show that, when the director acts as a facilitator or transformational leader [49] fostering the intrinsic motivation of collaborators; building bridges and improving communication while constantly managing the tension between objective goals (results) and subjective goals (learning and innovation), a “collective self” is seen to emerge. This collective self-fosters innovation and tries to balance cost-effectiveness with an investment in learning. In these cases the level of integration appears to be closer to “coordination” [1].

Finally, when the organisational context and culture seeks unity and trust in collective sense-making processes, the manager is able to act as a leader who is capable of orchestrating the three organisational goals towards purpose: results, learning and sense-making, promoting commitment, teamwork, empowering participants and promoting transcendent motivation. The emerging category “personal leadership” (Table 4) observed in some of the settings studied, especially in centre F, fits with the definition of system leadership according to Miller et al. [49]. This marriage of leadership, mission and culture in PCC seems to promote a level of integration that could be close to full integration, according to Leutz [1].

The ability to orchestrate the three different managerial roles over time requires the combination of different types of motivation and competences depending on the circumstances. The inductive process identified three types of managerial competences within the experiential learning category (Table 4), which enable organisational management to move towards consistency and equilibrium.

In terms of managerial skills, these relate to the achievement of objective results and include: having a vision of the organisation and its activity as patient-oriented, a proper management of resources, and negotiation and networking skills.

Interpersonal skills are subjective, including communication, conflict management, charisma, delegation, coaching, and teamwork.

Finally, intrapersonal competences appear to be related to sense-making through the intersubjective dimension. While some of these are oriented towards the external environment – proactivity, initiative, optimism, ambition, and personal management of time, information, and stress – others relate to the internal dimension: self-criticism, self-knowledge, and self-government. The latter are relevant in decision-making and require self-control, emotional balance, and integrity.

Table 8 summarises these experimental findings.

**Table 8:** Connections between organisational levers and management skills.

Level of integration observed	Organisational Dimensions Managed	Organisational Goals Sought	Organisational Performance Indicators	Director’s Role	Type of Leadership	Motivation styles observed by management	Competences observed by management

Linkage	Objective	Outcomes	Effectiveness	Manager	Transactional	Extrinsic	Technical
Coordination	Objective Subjective	Outcomes Innovation	Effectiveness Learning	Manager Facilitator	Transformational	Extrinsic Intrinsic	Technical Interpersonal
**Full** **Integration**	**Objective** **Intersubjective** **Subjective**	**Outcomes** **Sense-making** **innovation**	**Effectiveness** **Unity** **Learning**	**Manager** **Leader** **Facilitator**	**System**	**Extrinsic** **Transcendent** **Intrinsic**	**Technical** **intrapersonal** **Interpersonal**

In order to describe the analytical results on a more practical level for service-providers and managers, Table 9 shows the actions which could have the greatest positive impact on the process.

**Table 9:** Organizational actions undertaken.

Dimension	Organisational Goal	Organisational challenge	Actions

Intersubjective	Sensemaking in order to avoid fragmentation and opportunistic behavours	Establishing purpose and need	Review participatory organisational mission of service in order to promote: intrinsic dignity, well-being and personal autonomy
			Review organisational culture values; to build by consensus a shared values code and behaviour framework according to the new proposed paradigm: positive attitude, recognition of diversity, fluid and open communication, trust and collaboration
			Develop new leadership programmes according to above mission of service and cooperative culture
Subjective	Learning in order to innovate	Engaging and involving participants enabling them to contribute with their experience, knowledge and skills	Promote programs for empowerment and training for team work; mainly face-to-face; listening, assertiveness and empathy
			Work to see human diversity as a source of innovation
			Facilitate outside support for teams to learn to manage conflict, diversity and ambiguity
Objective	Results (outcomes) in order to be cost-effective	Working with processes and systems to adapt them to the proposed change	The implementation of small and stable teams is essential; no rotation at all
			Establish three teamwork spaces for: care provision, learning and sensemaking
			Review HR policies according to new values; establish the organisational performance indicators according to the new values
			Establish new professional roles; mainly, the dual role of operational managers and case manager “new power”
			Open IT systems for information flow; remove protocols, establish good practice guidelines
			Team meetings are a powerful tool for information flow and learning; participation should be mandatory in team work; establish a guide and training for effective meetings.

## Discussion

Reviews of evidence consistently highlight that many promising pilot experiments fail to become generalized and consolidated practices [8]. In fact, the innovation experience recognises management as either an enabler or barrier to successful development of integrated care [23,27,28,29,30]. Amelung et al. [50] note that “leadership is certainly one of the neglected topics in integrated care”.

Several scholars have stressed the pivotal role of leadership in promoting care integration, arguing that network and teamwork formation is not only a strategic, but also a leadership issue [51]. The experience of this case study illustrates how the director of a fully integrated care organisation needs to transcend the traditional role of a manager capable of handling the formal levers of the organisation. The generation of positive organisational context to promote decentralized and interdependent teamwork [52] designed to generate cost-effective and personalised care pathways, requires personal leadership to integrate participant’s diversity and promote collective thinking.

If, as managers, we are to truly recognise the intrinsic dignity of all participants in the care system, we must take into consideration that there are as many subjective realities as participants [53]. This implies managing the informal structure through a participative problem-solving oriented relationship style in order to see this diversity as a potential source of new knowledge and innovation and a powerful lever towards external adaptation and internal cohesion.

The results of this case study confirm that a person-centred organisational structure can help support co-operative culture by creating spaces for action-reflection within small and stable teams [54]. Such interdependent teams allow the fluid communication of information thanks to dual role operational managers and open IT systems which help reduce protocol [55] and promote participatory planning [56].

The importance of case managers, unit and team coordinators cannot be over-emphasised. It is their role to create the necessary conditions and spaces for action-reflection teamwork, for collective sense-making aimed towards satisfying people’s sense of purpose and individual needs. For this, experiential learning through cooperative teamwork is important in order to prioritise collective wellbeing over personal needs and short-term results [57]. It is therefore essential that management set up organisational policies and systems which support such participatory planning.

Leadership [49] emerges as a critical lever towards organisational integration of PCC. Strategic managers are the only players in a position to change the formal structures of the organisation and support bottom-up change from the top. Thus, the development of higher motivational quality to prioritise individual dignity and collective well-being is critical for managing the three dimensions in an integrated organisation. However, this involves a long and arduous work of personal commitment to prioritise the common good.

Unfortunately, this is something that not all people in these critical positions of management are willing to take on. In practice, the process generates different episodes of resistance to change especially when the base team push back against the change. When they are extrinsically incentivized, the first pattern, “fear of change” could emerge, due to the feeling of facing an unknown challenge alone, as well as the fear of failure or competition [16]. Consequently, affective team support is needed to minimize the negative impact of these reactions.

Thus, in accordance with the case model shown here, two conditions are proposed as necessary for a manager attempting to fully integrate PCC across his or her organisation. First, the ability to prioritise, and enable others to prioritise, transcendent motivation, i.e. dignity and the common good, over intrinsic-personal needs and extrinsic-organisational results in the decision-making process. And second, the acquisition of the technical, interpersonal and intrapersonal competences which will make it possible to implement these decisions. Both conditions constitute the essential bundle of skills which strategic managers would need in order to steer change [7,27,49] towards full organisational integration [1,23]. It is important to note that both these conditions refer to ‘soft’ skill-sets, which are beyond the formal control of protocol and regulations.

Finally, comments recorded in the interviews suggest that, when a strategic manager’s personal behaviour prioritizes the general interest, neither being opportunistic nor oriented towards short term results but rather responding to the emerging needs of participants by consistently managing the nine levers, participants develop a deep level of trust in themselves and in the process. On the one hand, they come to prioritise transcendent motives and on the other, they develop the confidence and competence (intrapersonal knowledge) needed to adapt to the changes which organisational integration requires.

## Lessons learned

- The nine Organisational levers: mission of service, cooperative culture, personal leadership, informal structure, relationship style, experiential learning, formal structure, policies and systems and participatory planning should be consistently managed to promote integration. This is difficult if they are out of the management range of a single integrated organization.
- Resistance and Fear emerge during the integration process when change is managed first from “Top-Down” logic. However innovation and certainty grow when change is managed from collective sense-making towards organisational purpose and needs.
- Necessity of understanding both the external organisational environment and internal domain of each participant that directly influence organisational performance. In spite of being beyond management control, they should be known in order to consistently adapt the nine organisational levers through teamwork in order to balance results, personal learning and sense-making.
- Strategic management: develop intentional and competence trust in practice by consistently managing the nine levers. This requires the ability to prioritise collective wellbeing (transcendent motivation) over personal interest (intrinsic motivation) or short term results (extrinsic motivation) and the development of technical, intrapersonal and interpersonal competences to implement them in line with PCC.
- Operational management: focus on the generation of practical participation, information flow, shared decision making, delegation and teamwork, promoting spaces for action and reflection towards wellbeing, learning and outcomes.

## Conclusions and limitations of the study

The main conclusion that can be drawn from this experimental study of strategic change management towards full integration [11] of PCC across a whole organisation [8] is the dynamic and subjective nature of the process. In this sense, the most effective way to improve the process of PCC integration is by encouraging managers to engage in action research of this nature. This type of organisational change requires changes in deeply held beliefs, values and behaviours in all stakeholders, patients, care providers and management. However, if carried through with sensitivity and dedication, the results of the process can be extremely positive for all involved, and even have a favourable impact on the bottom line. The process of engaging in qualitative action research, involving intensive, active listening to other stakeholders, tends to promote the development of the trusting relationships necessary to push through significant organisational change whilst assuring stakeholder engagement.

Clearly, the study described here does not claim to offer a blue-print for success for other organisations: every organisation and every context is different, and successful strategies for change will vary likewise. Further research is therefore needed to ‘test’ the experimental model developed here in different contexts and with different protagonists, to see if it is as helpful there as it has proved to be for Etxean Ondo.
